# Contact Lenses for Color Blindness

**DOI:** 10.1002/adhm.201800152

**Published:** 2018-04-26

**Authors:** Abdel‐Rahman Badawy, Muhammad Umair Hassan, Mohamed Elsherif, Zubair Ahmed, Ali K. Yetisen, Haider Butt

**Affiliations:** ^1^ School of Engineering University of Birmingham Edgbaston Birmingham B15 2TT UK; ^2^ Neuroscience and Ophthalmology Institute of Inflammation and Ageing University of Birmingham Edgbaston Birmingham B15 2TT UK; ^3^ School of Chemical Engineering University of Birmingham Edgbaston Birmingham B15 2TT UK

**Keywords:** color blindness, color vision deficiency, contact lenses, ocular diseases, vision correction

## Abstract

Color vision deficiency (color blindness) is an inherited genetic ocular disorder. While no cure for this disorder currently exists, several methods can be used to increase the color perception of those affected. One such method is the use of color filtering glasses which are based on Bragg filters. While these glasses are effective, they are high cost, bulky, and incompatible with other vision correction eyeglasses. In this work, a rhodamine derivative is incorporated in commercial contact lenses to filter out the specific wavelength bands (≈545–575 nm) to correct color vision blindness. The biocompatibility assessment of the dyed contact lenses in human corneal fibroblasts and human corneal epithelial cells shows no toxicity and cell viability remains at 99% after 72 h. This study demonstrates the potential of the dyed contact lenses in wavelength filtering and color vision deficiency management.

## 1 Introduction

**Figure 1:**
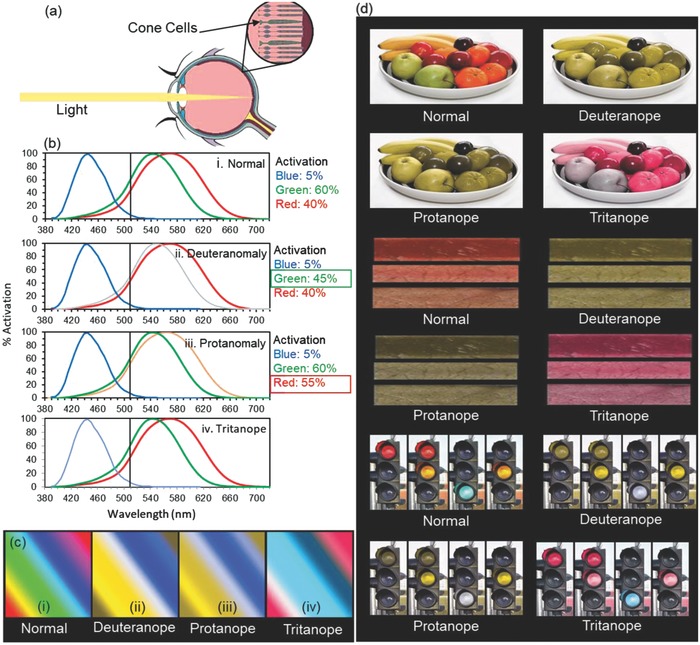
Color perception in CVD. a) The anatomy of the eye and cone cells. b) The activation percentages for the different types of CVD for 510 nm. c) A visual representation of what is seen by individuals looking at the same image with different color vision abilities. d) Colors perceived by individuals having different types of CVD.

Colors are used to aid in identifying universal color phases, emotions, illnesses, and the ripeness or freshness of produce.^1^, ^2^, ^3^ It can therefore lead to difficulties if these colors are not accurately perceived. Color vision deficiency (CVD) or color blindness is a common ocular disorder affecting, for example, an estimated 8% of males and 0.5% of women of Northern European descent.^3^, ^4^, ^5^, ^6^, ^7^, ^8^ It limits accurately distinguishing between specific colors depending on the type and intensity of the disorder.^3^, ^5^, ^7^, ^8^ CVD patients experience problems in work and everyday life when matching or discriminating between fine colors.^2^, ^9^, ^10^, ^11^, ^12^ Normal color vision is trichromatic;^11^ any color can be recreated by combining blue, red, and green, perceived by a cluster of photoreceptive cones at the back of the eye (**Figure** **1**a). These cones are divided into three groups, responsible for short wavelengths (blue), medium wavelengths (green), and long wavelengths (red).^13^ In normal vision, all three cones are present.^11^ When identifying a color, these cones function according to their corresponding activation thresholds.^14^ The combination of the different activation thresholds for the three types of cones is then processed by the brain and the corresponding color is perceived. When any of these cones are missing or defective, the brain receives incorrect information, leading to limited color perception.^4^ Three possible outcomes if one of the cones is defective are: (i) protanomaly, occurs when the cone responsible for red color is shifted to the left; (ii) deuteranomaly, the most common form of red‐green color deficiency occurs when the cone responsible for green is shifted to the right; and (iii) tritanomaly, the least common of the three occurs when the cone responsible for blue is displaced (Figure 1b–d).^15^

Dichromatism, a condition in which one type of cone is completely missing, is divided into three subgroups—protanopia, deuteranopia, and tritanopia, that is, when the eye lacks the cones responsible for red, green, and blue wavelengths, respectively.^11^, ^15^ Protanopia, protanomoly, deuteranopia, and deuteranomaly are collectively referred as red‐green color vision deficiency.^5^ Tritanopia and tritanomly are both rarer, affecting ≈1% of males and 0.03% of women.^5^, ^13^ The rarest form so called monochromatism occurs when the eye has either no cones at all or has only the cone responsible for blue color, causing a complete lack of the ability to perceive color.^13^ CVD patients develop adaptive strategies,^4^, ^16^ for example, those having dichromatism displayed normal colored vision under certain test conditions validating the possibility of exploiting external factors for increased color perception.^17^ This suggested the possibility to deceive the brain into perceiving wavelengths, which the eyes are unable to accurately detect.

While no cure for CVD currently exists, several management techniques can be used to increase the color perception of affected patients.^5^ One such method is the use of tinted spectacles.^5^ The use of colored filters has been investigated to correct CVD.^9^ While color discrimination was improved, it did not result in complete normal color vision.^18^ Corrective glasses have been utilized to filter out a narrow range of wavelengths (≈545–575 nm) responsible for the largest confusion between the affected cones by using a multinotch filter.^18^, ^19^ This method has been experimentally shown to improve color vision.^20^ Several companies (e.g., Enchroma) offer products for CVD patients (Figure S1, Supporting Information).^21^, ^22^, ^23^ Multiple layers of transparent materials are coated on glasses to filter out a band of certain wavelengths minimizing the overlap between blue and green, and green and red colors transmitted to the photoreceptive cones in the eye.^24^ Existing color‐corrective glasses are high cost, bulky, and incompatible with other vision correction lenses. Here, a cost‐effective contact lens was created for CVD management. The possibility of using contact lenses for CVD management is of interest particularly because of their ability to provide the entire corrected field of view and avoid obstructions from uncorrected peripheral vision inevitable in the case of glasses.

## 2 Results and Discussion

**Figure 2:**
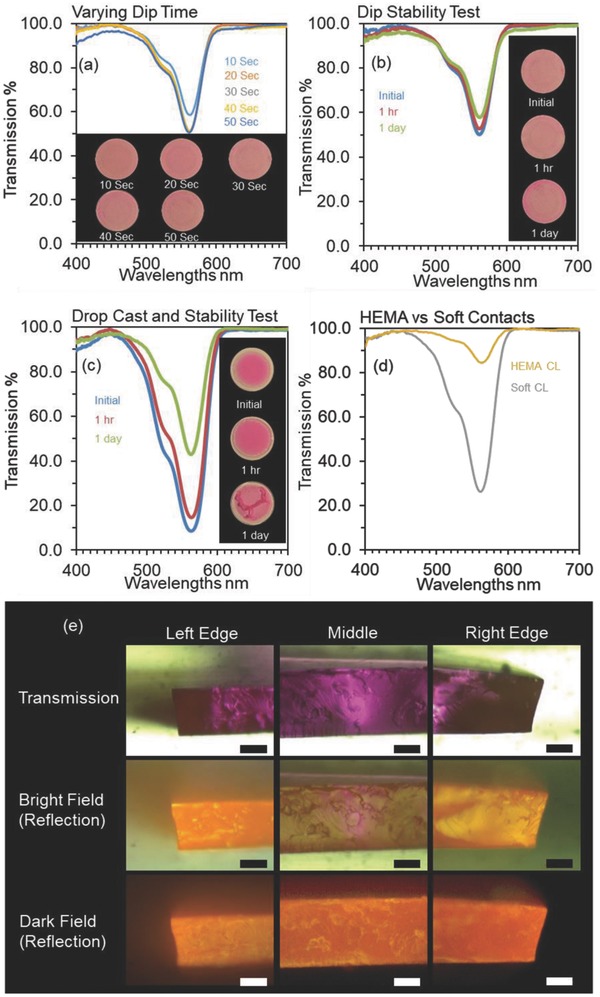
Transmission spectra of dyed contact lenses. a) The effect on transmission by varying dip time. The inset shows photographic images of the color of the lenses at the specified dip times. b) The dip stability test showing the change in transmission with respect to time. c) The drop‐cast method and its stability with inset showing the photographs taken after specified intervals of time. d) Transmission spectra of pHEMA and commercial soft contact lenses when dipped into the dye. e) The microscopic images of the cross sections taken under different viewing conditions. Scale bars = 100 µm.

The stabilities of the dip and drop methods (explained in Experimental Section) were compared (**Figure** **2**). All dipping processes were 30 s. Despite the dip method does not block the light band (545–575 nm) as much as the drop method, it is more suitable in terms of stability and CVD management. As over 24 h, the transmission dip decreased only 7% for the dip‐coated lens compared to 42% for the drop‐cast lens (Figure 2b,c). This may be attributed to the contact lens not fully absorbing the dye—most of the dye dried out on the surface without diffusing into the lens (Section S2, Supporting Information). This caused the excess surface dye to dissolve more quickly when dipped in the test solution, varying the transmission properties of the contact lens at a faster rate. In comparison, the dip method provided increased immobilization of the dye in the contact lens matrix. Varying the dip time at *t* > 20 s had a negligible effect on the transmission spectra because of the saturation of the bulk (Figure 2a). The absorption saturation occurs at *t* > 20 s of the dipping time. The lack of any excess surface dye decreased the dispersion rate of the dye within the lens and therefore decreased the rate of transmission increase (Figure 2b). The dip method was also found to provide improved immobilization of the dye when used on pHEMA contact lenses. However, when compared to the soft contact lens, the pHEMA contact lens showed lower ability to absorb the dye. Due to this poor absorption capability, its transmission (86%) dip was smaller than that of the soft contacts (26%) (Figure 2d). As the absorbance of the pHEMA contact lenses was low, the 23 wt% dye solution was used for this experiment. Soft contact lenses were used in subsequent experiments due to their superior absorption of the dye.

Cross‐sectional images of the dipped soft contact lens were used to evaluate the permeation of the dye through the contact lens (Figure 2e). Furthermore, the distribution of the dye throughout the contact lens was reasonably uniform. Dark‐field and bright‐field reflection and transmission images were obtained to evaluate the edges, surface, and bulk of the dyed contact lens. The images displayed a uniform distribution without any lumping of the dye within the contact lens and did not indicate any loss of integrity of the contact lens due to the dipping method.

**Figure 3:**
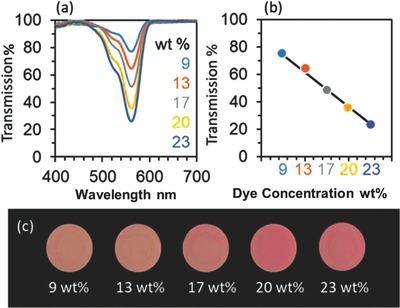
Optimization of rhodamine dye concentrations. a) The effect of varying dip concentrations. b) The relationship between dye concentration used and absorption. c) Images of the contact lenses dipped in different dye concentrations.

With the application method evaluated, the different concentrations of the dye were tested (**Figure** **3**a–c). The higher concentration dyes provided a deeper transmission dip, for 23 and 9% solutions the transmission dips of the dyed lenses were 72 and 21%, respectively. By plotting the concentrations against the transmission dips, a linear trend between the two was found. This trend provided customizability for the transmission dip of the dye and allowed rational optimization of the dye concentration.

**Figure 4:**
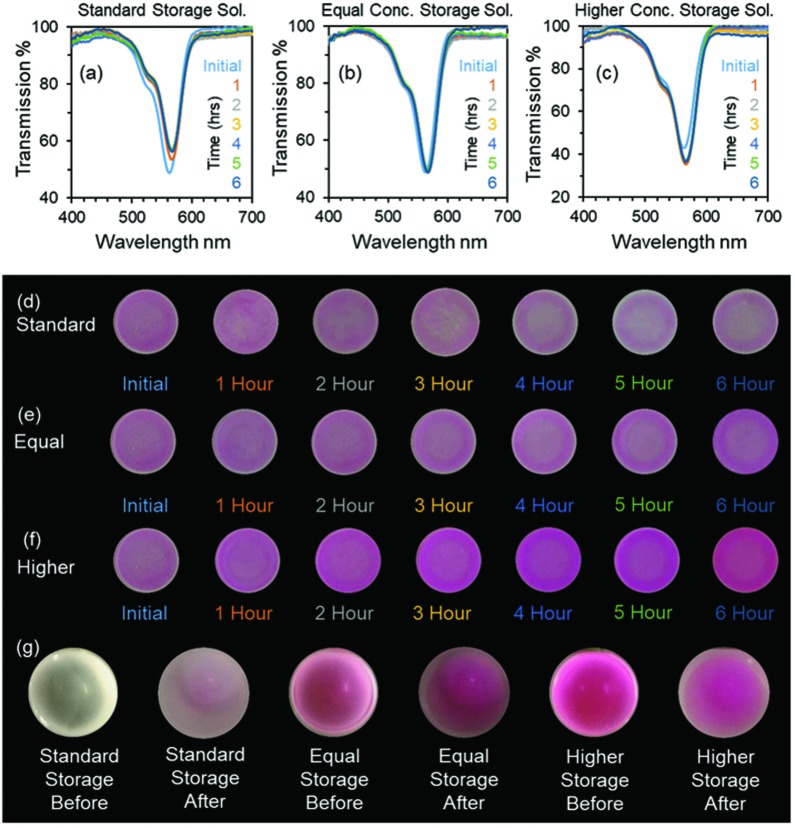
Dye diffusion from the contact lenses. The effect of time on the lenses when dipped into a storage solution with a) no added dye, b) 20 µL of equal concentration dye, and c) 20 µL of concentration dye (20 wt%). d–f) Images of the contact lenses corresponding to the graphs shown in (a)–(c), respectively. g) Change in the color of the storage solution at the beginning and end of each experiment.

As contact lenses were stored in a saline solution, the reaction between the dyed contact lenses and the storage solution was investigated (**Figure** **4**). The dye showed an initial weak diffusion into the storage solution after submersion within the solution as no sealing or cross‐linking technique was used to secure the dye within the contact lens. After 2 h, the change in transmission stopped (Figure 4a). When the concentration of the dye within the contact lens and its concentration within the storage solution were balanced, the dye diffusion ceased. To decrease this diffusion, 17 wt% dye (20 µL) was added to the storage solution to decrease the concentration gradient between the contact lens and the saline solution, where no diffusion was recorded after 6 h of submersion. Usage of higher concentration of dye (20 µL of 20 wt%) inversed the initial concentration gradient and increased the transmission dip of the contact lens by ≈9% within the first 2 h.

**Figure 5:**
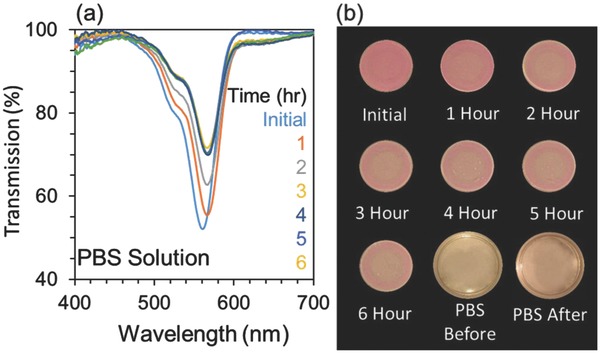
Dyed contact lens in PBS solution. a) The change in transmission peaks of the dyed contact lens when dipped in PBS solution as a function of time. b) Photographs of the change in the color of the dyed contact lens when dipped PBS solution. The change in the color of PBS solution is shown in the last two images.

As the dyed contact lens would come in contact with the eye, a phosphate buffered saline (PBS) solution was used to simulate the tear behavior (**Figure** **5**). The contact lens sample showed an initial maximum transmission dip of 52% which increased to 56 and 63% after first and second h, respectively. The transmission spectra recorded after 6 h ≤ *t* ≤ 3 h, showed almost identical transmission dip at ≈70%. The dye in the contact lens diffused into the PBS solution indicating the need for better immobilization of the dye within the contact lens. The contact lenses were kept in the storage solution for one month, where no apparent degradation was observed.

The validity of the dyed contact lenses as a possible CVD management technique was evaluated. The filtering mechanism was based on the stoppage of the increased overlapped band, that is, the common region of green and red wavelength bands perceived by the two types of corresponding optical cones. The dye effectively absorbed (blocked) this wavelength band. Therefore, the suppressed transmission of this band through as‐made contact lenses did not trigger the optical cones in common (green‐red) band region. As a result, enhanced color perception or CVD management was achieved. A survey consisting of both normal color sighted (NCS) individuals and individuals suffering from red‐green color deficiency was used to test the effects of the dyed contacts (Figure S3, Supporting Information). When asked to compare the numbers visible on printed copies of the Ishihara test, all of the NCS participants detected a varying range improvement of the colors for different screens. In comparison, the levels of improvement acknowledged by the participants affected by CVD varied substantially. However, all participants identified the improvement to the colors of their surrounding when looking through the contact lens, which concurs with the results of the commercial color vision correction glasses.^18^ The main complication when performing this survey was ensuring the color perception capabilities of those tested. Several of the self‐declared color deficient participants were unaware of their CVD condition. Additionally, many of those tested also suffered from slight blue/purple CVD as well as red‐green CVD. As the lenses only targeted the wavelengths concerning red‐green CVD, its effectiveness on those individuals with blue/purple CVD would be greatly reduced. Furthermore, as screening processes to identify CVD is no longer compulsory in schools,^4^ some individuals with normal color vision could believe that they suffer from CVD.

**Figure 6:**
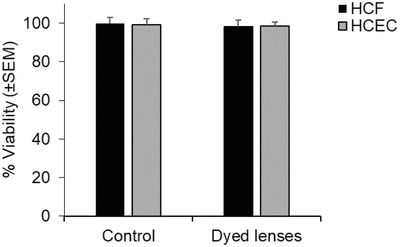
Cell viability after 72 h exposure to dyed lenses. 5 × 10^3^ HCF and HCEC were incubated in six‐well tissue culture plates in DMEM + 10% FCS for 72 h at 37 °C and 5% CO_2_. The MTT assay reagent (0.5 mg mL^−1^) was added to each well and the samples were incubated for 4 h in dark. The medium was then removed and precipitates were resuspended in DMSO and measured on a plate reader set at 570 nm. Each experiment was performed in triplicates.

Dyes are capable of absorbing narrow ranges of wavelength that can be controlled by altering their chemical structure.^25^ While both dyes and Bragg mirrors can be synthesized to be highly selective light filters, dye processing does not require complex preparation methods. This method could potentially be used for both glass spectacles and contact lenses at low cost. Since the dyed contact lenses are to be used in the eye, the 3‐(4,5‐dimethylthiazol‐2‐yl)‐2,5‐diphenyltetrazolium bromide (MTT) assay was used to assess potential toxicity to human corneal fibroblast (HCF) and human corneal epithelial cells (HCEC) after 72 h in culture. No toxicity of the dyed contact lenses was observed when compared to untreated cells and cell viability remained at ≈99% after 72 h, suggesting that the dyed contact lenses are nontoxic to human corneal cells (**Figure** **6**). During our biocompatibility testing, no dye accumulation was observed in the cells. Although there is a potential for the dye to be taken up by cells of the eye, this may not be a problem since live cells will be able to exclude the dye, such as propidium iodide (used by ophthalmologists), which is a nonpermanent dye. However, to avoid possible leakage in contact lenses, the dye could be cross‐linked to the lens matrix. Encapsulation is also an alternate stabilizing method to immobilize the dye.

## 3 Conclusion

A color filtering contact lens was successfully fabricated by submerging them in Atto 565 dye for 30 s. Furthermore, the concentration of the dye used could be controlled to accurately provide a high level of customization of the process. Due to its customizability, low cost of the dye, and the ease of the production process, these dyed contact lenses can be promising for CVD management. The main drawback of the process was the dye diffusion in the PBS solution. This diffusion may be controlled using covalent bonding to contact lens polymer backbone and incubation in hydrophobic preservation materials or oils. Furthermore, the dyes were nontoxic to human corneal fibroblasts and human corneal epithelial cells. The results of the survey verified that dye tinted contact lenses can be used to slightly improve the color perception of both those affected by color vision deficiency and those without. However, this improvement may not allow those affected with CVD to have comparable color vision as those with normal color vision. Further trials are required to investigate the ideal peak absorption percentage to optimize this enhancement. This experiment had several considerations. One important consideration is that the flow of the tear solution on the eye is ≈1.0 µL min^−1^, which is substantially lower than the 150 µL solution of PBS used. Therefore, the true leakage within the eye even without binding is expected to be lower than that recorded. Several solutions can be considered while preparing such lenses for clinical trials or practical use including incorporation of encapsulation method or crosslinking processes during the manufacturing stage or when the dye is applied to them postproduction. The fabricated contact lens is a step forward in the development of wearable devices for the management of color vision deficiency.

## 4 Experimental Section

**Figure 7:**
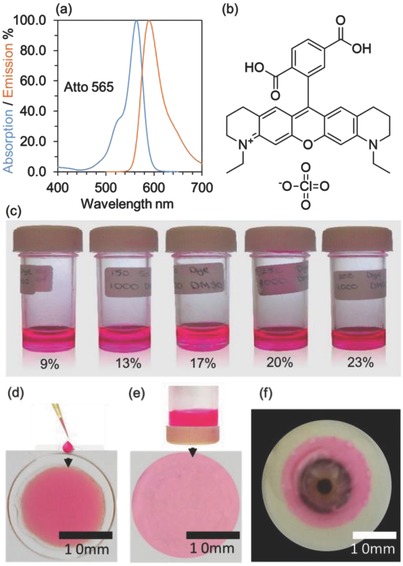
Rhodamine derivative (Atto 565) and its incorporation into contact lenses. a) The absorption and emission spectra of the dye. b) The chemical structure of the Atto 565 dye. Schematics of the d) drop method and e) dip method. f) The dyed contact lens on an artificial eye model.

A fluorescent rhodamine dye, 6‐(2,5‐dicarboxyphenyl)‐1,11‐diethyl‐3,4,8,9,10,11‐hexahydro‐2H‐pyrano[3,2‐g:5,6‐g′]diquinolin‐1‐ium (Atto 565, Sigma Aldrich) was used for the wavelength filtering properties of contact lenses. The dye offers an absorption band between ≈545 and 575 nm with a peak absorption occurring at ≈565 nm (**Figure** **7**a,b). It is moderately hydrophilic and has high photostability. Dye solutions were prepared using dimethyl sulfoxide (DMSO) to obtain 9, 13, 17, 20, and 23 wt% concentrations.

The drop and dip methods were used to apply the dye on homemade poly(2‐hydroxyethyl methacrylate) (pHEMA) lenses and commercial silicone based 1 d contact lenses (Acuvue Moist) (Figure 2d,e). Commercial lenses were selected for further optimization. The drop method entailed directly casting a drop of the dye (20 µL) on the lens surface, ensuring that the center of the lens was evenly covered (Figure 7d). The dipping method involved dipping the contact lens into the dye solutions for 1 min. To prevent contact lens folding, it was placed onto the inside face of a surface. This surface was then inverted allowing the dye to encompass the contact lens (Figure 7e). The distribution of the dye across the lens was also found to be uniform using this method. Cross‐sectional microscopic images of these samples were taken to analyze the permeability of the dye in the contact lens. The optimal dip time was then investigated by submerging the contacts into the dye solution (17 wt%) for 10, 20, 30, 40, and 50 s. The lenses were then scanned using an optical spectrophotometer (Ocean Optics, USB 2000) and the resulting transmission profiles were compared. Drop‐cast and coating techniques were compared using spectroscopy by recording their absorption spectra at different time intervals after applying the dye. For dip processed lenses, 17 and 25 wt% dye solutions (20 µL) were added to the storage solution (150 µL), and hourly readings were recorded. Additionally, the contact lens was submerged within daily contact lens saline storage solution (50 µL). Similarly, the reaction between the dyed contact lens and a phosphate‐buffered artificial tear solution (150 µL) was analyzed. As some contact lens wearers find the need to hydrate their eyes using artificial tears while wearing the contacts, the dyed contacts were also immersed within 150 µL of artificial tears. Hourly readings were recorded for both tests.

To analyze the effectiveness of the dyed contact lenses, a survey was performed under the guidelines and permission of University of Birmingham Ethical Review. Individuals with normal color vision and those having red‐green color vision deficiency were asked to identify several numbers taken from the different standard Ishihara test slides. These individuals were then asked to look through the dyed contact lens—which was applied on a glass slide—and note whether there were any improvements to the colors or the clarity of the number. They were then asked to restate the number that they observed. Finally, they were asked to observe their surroundings and note whether any adequate improvement could be noted.

To investigate potential toxicity of the dyed contact lenses to cells in the eye, HCFs were expanded in Dulbecco's modified Eagle's medium (DMEM) and 10% fetal calf serum (FCS) (Invitrogen), 5 × 10^3^ cells were plated in six‐well plates and covered with 2 mL of DMEM + 10% FCS. Contact lenses were immersed into the culture medium and left in contact with cells for 72 h at 37 °C and 5% CO_2_. HCECs (Millipore) were expanded in EpiGRO medium (Millipore) and 5 × 10^3^ cells were inoculated in six‐well plates, covered with 2 mL of EpiGRO and exposed to contact lenses as described above for HCF. After 72 h, supernatants were removed and cells were washed in PBS and the MTT assay was performed according to the manufacturer's instructions (R&D Systems, Watford, UK). Briefly, MTT reagent (100 µL, 0.5 mg mL^−1^) was added to each well and incubated for 4 h before incubation for a further 2 h in detergent reagent (100 µL). The absorbances of the resultant solutions were read at 570 nm and absorbance units calculated from triplicate readings after subtraction of blank wells (culture media only).

## Conflict of Interest

The authors declare no conflict of interest.

## Supporting information

Supplementary
