# Supplementary material for: Contact Lenses for Color Blindness
Source: Adv Healthc Mater. 2018 Apr 26;7(12):1800152. doi: 10.1002/adhm.201800152 (PMC6691754; doi:10.1002/adhm.201800152)
Supplement: Supplementary file 1 — Supplementary [file ADHM-7-na-s001.pdf]

Copyright WILEY-VCH Verlag GmbH & Co. KGaA, 69469 Weinheim, Germany, 2018.

ADVANCED HEALTHCARE MATERIALS

Supporting Information

for *Adv. Healthcare Mater.*, DOI: 10.1002/adhm.201800152

Contact Lenses for Color Blindness

*Abdel-Rahman Badawy, Muhammad Umair Hassan, Mohamed Elsherif, Zubair Ahmed, Ali K. Yetisen, and Haider Butt**

SUPPORTING INFORMATION

Contact Lenses for Color Blindness

Abdel-Rahman Badawy[a], *Muhammad Umair Hassan*[a], *Mohamed Elsherif*[a], *Zubair Ahmed*[b], *Ali K. Yetisen*,[c] *Haider Butt*[a*]

[a]School of Engineering, University of Birmingham, Edgbaston, Birmingham, B15 2TT, UK

[b]Neuroscience and Ophthalmology, Institute of Inflammation and Ageing, University of Birmingham, Edgbaston, Birmingham, B15 2TT, UK

[c]School of Chemical Engineering, University of Birmingham, Edgbaston, Birmingham, B15 2TT, UK

Keywords: Contact lenses; ocular diseases; color blindness; rhodamine; vision correction

[*]Corresponding Author's Email: h.butt@bham.ac.uk

S1. The transmission of selective wavelength bands is minimized in order to make CVD management glasses (**Figure S1**).

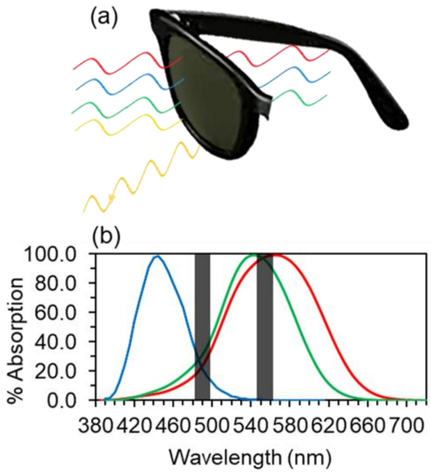

Figure S1. a) concept diagram of wavelengths filtering for color blinds using color enhancing glasses. **b)** the wavelengths filtered by the color enhancing glasses.

S2. The uniformity of the diffused dye across the thickness of the contact lens is dependent on the technique used to dye to the lens. The drop-cast method gave a non-uniform distribution of the dye (**Figure S2**).

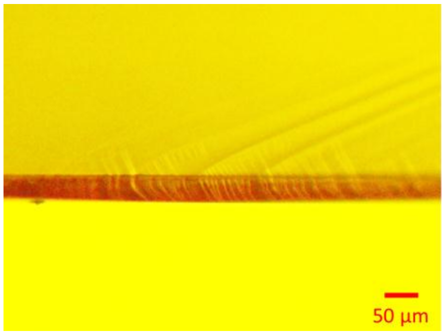

Figure S2. Cross-sectional image of a drop-cast contact lens. The image illustrates the non-uniformity of the dye across the cross-section of as-made contact lenses.

S3. The validity of the dyed contact lenses as a possible management technique was also explored. A quick survey consisting of both normal color sighted (NCS) individuals and

individuals suffering from red-green color deficiency were used to test the effects of the dyed contacts. When asked to compare the numbers visible on printed copies of the Ishihara test, all of the NCS participants noted that the colors appeared sharper for the plates, helping them see the correct number clearer. As for those affected by CVD, the results were more varied. While no improvement was found for most plates, several individuals noted a slight improvement for a few plates. However, the noted improvement did not allow them to see the correct number but made their previously perceived number slightly sharper. This is in line with what was expected when compared to the Enchroma glasses. In comparison, all of those affected with CVD noted a slight improvement when observing their surroundings, while the improvement noted by those with normal color vision varied. However, these introduce the possibility of using the contact lenses as a color enhancer for normal color-sighted people as well. Unfortunately, a comparison between the effectiveness of the contact lenses and the Enchroma glasses was not possible as a pair of Enchroma glasses were not available for the survey.

The main complication when performing this survey was ensuring the color perception capabilities of those tested. Several of the self-declared color deficient participants were unaware of the type of CVD that they were inflicted with. As a result, the effectiveness of the lenses for the different CVD types could not be compared. Additionally, many of those tested also suffered from slight blue-yellow CVD as well as red-green CVD. As the lenses only targeted the wavelengths concerning red-green CVD, its effectiveness on those individuals would be greatly reduced. Furthermore, as screening processes to identify CVD are no longer compulsory in schools (Albany-Ward 2015), some individuals with normal color vision could believe that they suffer from CVD when they do not. These complications introduced a level of uncertainty in the results. Therefore, a more thorough clinical trial should be used to verify these results.

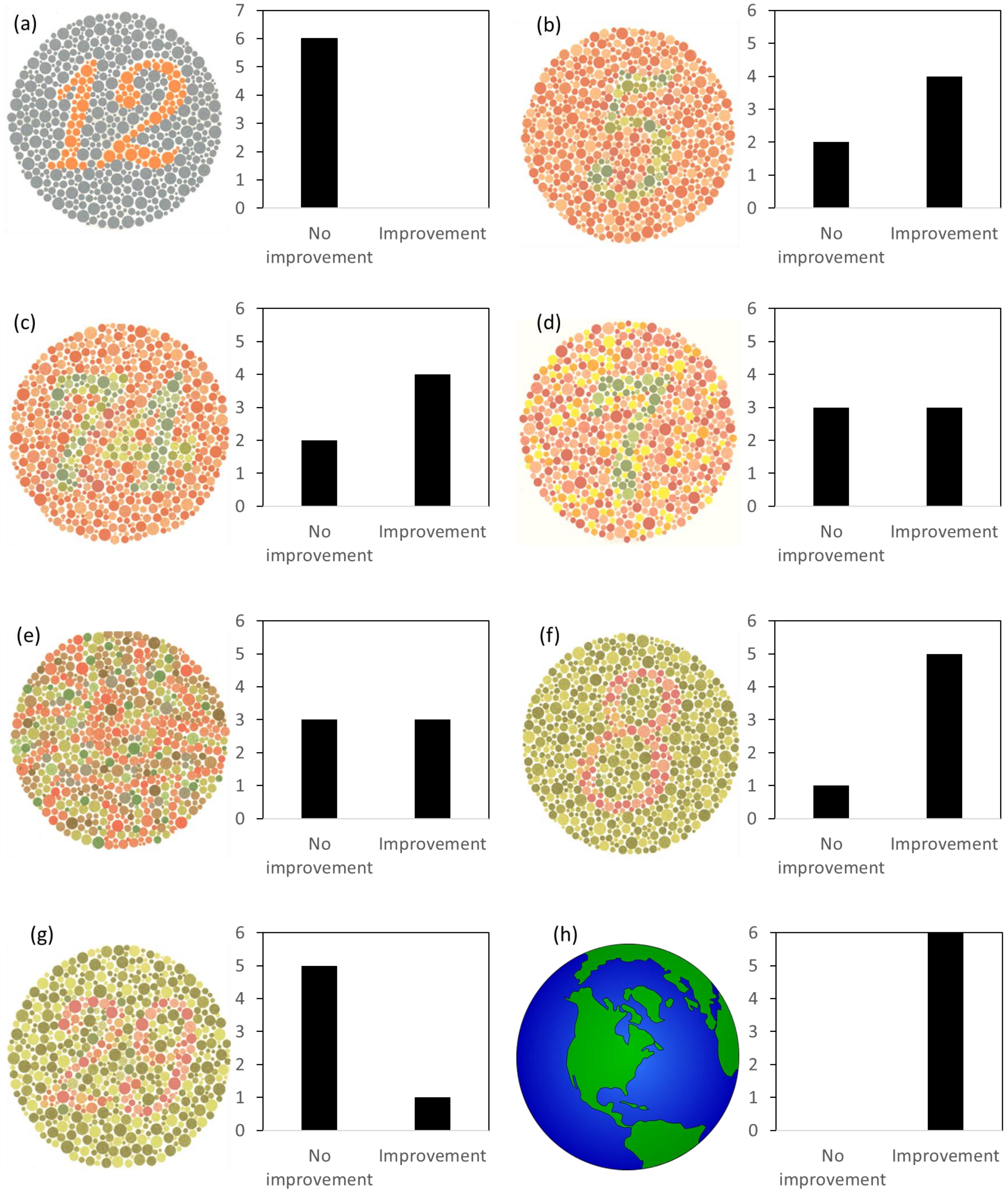

Figure S3. Results from the survey conducted to carry out an initial assessment of the effectiveness of dye-based solution for CVD management.
